# Substrate Utilisation and Energy Metabolism in Non-Growing *Campylobacter jejuni* M1cam

**DOI:** 10.3390/microorganisms10071355

**Published:** 2022-07-05

**Authors:** Emily Stoakes, George M. Savva, Ruby Coates, Noemi Tejera, Mark G. Poolman, Andrew J. Grant, John Wain, Dipali Singh

**Affiliations:** 1 Department of Veterinary Medicine, University of Cambridge, Cambridge CB3 0ES, UK; eas202@cam.ac.uk (E.S.); rc715@cam.ac.uk (R.C.); ajg60@cam.ac.uk (A.J.G.); 2 Quadram Institute Bioscience, Norwich Research Park, Norwich NR4 7UQ, UK; george.savva@quadram.ac.uk (G.M.S.); noemi.tejera-hernandez@quadram.ac.uk (N.T.); 3 Cell System Modelling Group, Oxford Brookes University, Oxford OX3 0BP, UK; mgpoolman@brookes.ac.uk

**Keywords:** *Campylobacter jejuni*, substrate utilisation, cell survival, energy metabolism, metabolic network

## Abstract

*Campylobacter jejuni*, the major cause of bacterial foodborne illness, is also a fastidious organism that requires strict growth requirements in the laboratory. Our aim was to study substrate utilisation and energy metabolism in non-growing *C. jejuni* to investigate the ability of these bacteria to survive so effectively in the food chain. We integrated phenotypic microarrays and genome-scale metabolic modelling (GSM) to investigate the survival of *C. jejuni* on 95 substrates. We further investigated the underlying metabolic re-adjustment associated with varying energy demands on each substrate. We identified amino acids, organic acids and H${}_{2}$, as single substrates supporting survival without growth. We identified several different mechanisms, which were used alone or in combination, for ATP production: substrate-level phosphorylation via acetate kinase, the TCA cycle, and oxidative phosphorylation via the electron transport chain that utilised alternative electron donors and acceptors. The benefit of ATP production through each of these mechanisms was associated with the cost of enzyme investment, nutrient availability and/or O${}_{2}$ utilisation. *C. jejuni* can utilise a wide range of substrates as energy sources, including organic acids commonly used for marination or preservation of ingredients, which might contribute to the success of their survival in changing environments.

## 1. Introduction

*Campylobacter* spp. are the leading cause of acute bacterial gastroenteritis worldwide. Clinically, the scale of campylobacterosis, as well as the impact on the individual, must be considered. Although the debilitating symptoms of acute infection, such as diarrhoea and fever are usually self-limiting, severe post-infection sequelae also occur, including reactive arthritis (1–13%), Guillain Barré syndrome (0.001%), and inflammatory bowel disease [1,2]. Given the number of cases, these percentages represent large numbers of patients, and the estimated global cost of campylobacteriosis is several billions of US dollars annually; in the UK alone the cost to the economy is around GBP 0.71 billion per year [3].

*Campylobacter jejuni*, the most frequently reported species associated with human campylobacteriosis [4], is a fastidious organism that is difficult to grow under laboratory conditions. Different species require different conditions that include temperatures of 37 to 42 °C and specific atmospheric conditions, including a gas mix of 5–10% O${}_{2}$, 5–10% CO${}_{2}$ and 80–85% N_2_, with some strains also requiring H${}_{2}$ [5]. Individual isolates, including different strains of *C. jejuni*, also show varied substrate auxotrophies, making the investigation of nutrient requirements for growth necessary but difficult [6,7,8]. Despite these strict growth requirements, *C. jejuni* can be isolated from a wide range of environmental niches, including water, soil, food, and milk [4]. This suggests that *Campylobacter* as a group of organisms show a great deal of metabolic flexibility to enable the different nutrients present as substrates in this wide range of niches to be used. Substrate utilisation in microbes is often associated with growth; however, this is not always the case [9,10], as energy is required even in the non-growing, or dormant state [11], which has long been recognised as a microbial survival strategy [12]. The success of *Campylobacter* as a foodborne human pathogen depends on the ability to survive in the food chain, and so it is important that we understand metabolism without growth for this major foodborne pathogen.

**Figure 1:**
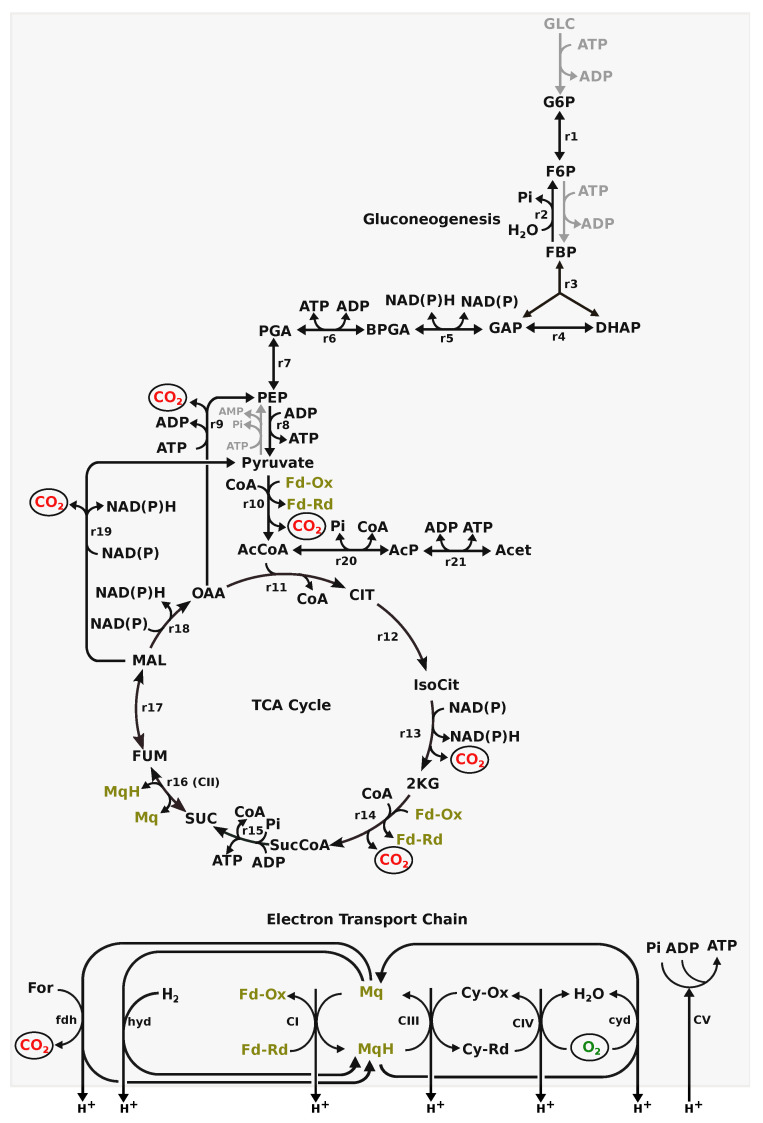
Central metabolism in *C. jejuni* M1cam. r1-r9: reactions involved in gluconeogenesis; r10: pyruvate:ferredoxin oxidoreductase (PFOR); r11-r18: TCA cycle; r19: malic enzyme; r20: phosphate acetyltransferase; r21: acetate kinase; fdh: membrane-bound formate dehydrogenase; hyd: membrane-bound hydrogenase; C1-CV: complex I to V in the ETC; cyd: cytochrome quinol oxidase; Mq: menaquinone; MqH: reduced menaquinone; Cy-Ox: oxidised cytochrome C; Cy-Rd: reduced cytochrome C; greyed out reactions are absent.

For *Campylobacter* spp., metabolic activity during growth, in particular substrate utilisation and energy metabolism, is well studied [13,14]. Access to substrates is not fully understood and there is a lack of transporters for many small carbohydrates, such as glucose [15,16,17], with the exception of L-fucose [14,18,19]. As shown in Figure 1, the Embden–Meyerhof–Parnas (EMP) pathway lacks two enzymes, glucokinase and phosphofructokinase (greyed out reactions in Figure 1). All the enzymes for gluconeogenesis, except phosphoenolpyruvate synthase (greyed out), are present. Utilisation of hexoses, therefore, is not optimised and the metabolic pathway flux in central metabolism cannot be investigated using the metabolic map for bacteria (such as *Escherichia coli*), where the EMP is complete. In the tricarboxylic acid (TCA) cycle, there are many oxygen-labile iron-sulphur-cluster-containing enzymes, such as pyruvate:acceptor oxidoreductase (PFOR) (r10) and 2-oxoglutarate: acceptor oxidoreductase (KGOR) (r14) contributing to *C. jejuni* sensitivity to O${}_{2}$, as molecular oxygen or reactive oxygen species can damage the iron-sulfur complexes [20]. Both these enzymes, PFOR and KGOR, have preference for ferredoxin/flavodoxin as an electron acceptor over NAD [21,22]. This preference for ferredoxin/flavodoxin utilising enzymes in the TCA cycle is suggested to establish a direct link for ATP production via the respiratory electron transport chain (ETC) [23]. Unlike the classic *Escherichia coli*, Complex I (CI in Figure 1), in the ETC of *C. jejuni*, flavodoxin/ferredoxin is preferred as an electron donor over NADH [24]. The ETC itself also shows flexibility. The *C. jejuni* M1cam genome encodes two types of terminal oxidase—cbb3-type cytochrome-c-oxidase (CIV) and a cytochrome bd-type quinol oxidase (cyd) [25]— that can utilise O${}_{2}$ as the terminal electron acceptor during aerobic respiration. The genome also encodes a range of alternative terminal reductases, such as nitrate reductase and fumarate reductase (r16 or CII), that allow the use of electron acceptors other than O${}_{2}$ to support anaerobic respiration [26,27,28]. Furthermore, the presence of a periplasmic formate dehydrogenase (fdh) and several hydrogenase (hyd) reactions explains how *C. jejuni* can utilise formate and H${}_{2}$, respectively, as electron donors [29,30]. Finally, the presence of phosphate acetyl-transferase and acetate kinase (r20 and r21, respectively, in Figure 1) could facilitate energy production through substrate-level phosphorylation. This all supports experimental observations that the preferred carbon and energy sources for the *Campylobacter* spp. studied are amino acids, organic acids, and TCA cycle intermediates, such as serine, proline, lactate, pyruvate, and succinate [28,31,32].

Taken together this represents a remarkable range of alternative routes for energy metabolism, possibly providing the biological advantage of being able to meet energy demands in a wide range of environments, specifically for survival, but not growth, in the food chain. Several substrates required for the growth (an increase in biomass) of *C. jejuni* [8] are likely absent from the food chain, but the flexibility of *C. jejuni* metabolism means that electron donors and acceptors for energy metabolism are present, allowing this persistent pathogen to survive in the food chain [33,34]. However, whether the use of different electron acceptors and donors to meet varying energy demands for survival without growth occurs metabolically remains largely untested experimentally.

In this study, we investigated the metabolic activity linked to energy (ATP) production experimentally using conditions under which biomass could not be produced, with different single substrates as the potential energy source. The data were then explored using a strain-specific in silico, genome-scale metabolic model (GSM) (see Appendix A for further details), to evaluate the mechanisms by which *C. jejuni* M1cam generates ATP for non-growth-based survival. Using the GSM, we further investigated possible metabolic routes for ATP production from each single substrate by limiting the substrate uptake flux whilst increasing ATP demand; this was to see how the network re-adjusted for increased efficiency of ATP production.

## 2. Materials and Methods

### 2.1. Bacterial Strains and Growth Conditions

*C. jejuni* M1cam (M1 isolate [35]) was routinely cultured on Mueller–Hinton (MH) agar (Merck Life Science UK Limited, Dorset, UK) at 42 °C under microaerophilic conditions of 5% O${}_{2}$, 5% CO${}_{2}$, 5% H${}_{2}$, 85% N_2_ in an M95 variable atmosphere workstation (Don Whitley Scientific, Bingley, UK). Bacterial stocks were stored at −80 °C in MicroBank tubes (Pro-Lab Diagnostics, Birkenhead, UK). For each experiment and replicate, bacteria were always grown from freezer stocks onto fresh MH agar plates for 48 h and then restreaked and grown for a further 16 h before use.

### 2.2. Biolog Assay

Biolog Phenotypic microarrays (PM) are preconfigured 96-well plates containing different classes of chemical compounds. Biolog redox dye (tetrazolium dye) is reduced by reductants such as NADH or NADPH to produce a purple colour indicating that the cells are metabolically active and can utilise the specific substrate for respiration. In this study, we used the PM1 plate (that contains substrates such as amino acids, TCA cycle intermediates, and organic acids). *C. jejuni* M1cam was prepared using the recommended protocol from Biolog with the following modifications: The 12X PM additive solution was made without BSA, filter-sterilized and stored at 4 °C before use. To avoid any effect of BSA on substrate utilisation and respiration, it was removed for all experiments. Three biological repeats were performed.

Bacteria were scraped off agar plates using sterile PBS (pH 7.2), and centrifuged at 5000× *g* at 4 °C for 5 min. Bacteria were then washed a total of 3 times using sterile PBS (pH 7.2), pelleting after each wash, and finally resuspended in 1mL of IF-0α GN/GP. The bacterial suspension was then diluted to an OD${}_{605\mathrm{nm}}$ of 0.4 with IF-0α GN/GP and mixed with the recommended amounts of water, 12× PM additive, IF-0α GN/GP and dye mix. A quantity of 100 μL of this mixture was then added to each well of the Biolog PM1 plate. From this starting plate, absorbance (OD${}_{605\mathrm{nm}}$) was measured using an Absorbance 96 plate reader from Byonoy (Hamburg, Germany). Bacteria were taken from the starting inoculum and were counted using serial dilutions. Dilutions were plated onto MH agar plates and colony-forming units (CFU) were counted after 24 h under microaerophilic conditions at 42 °C. Alongside each plate, an abiotic control plate was set up using all components except for the bacteria. The abiotic control plates were used to identify carbon sources that could indicate false-positive results due to the auto-reduction of the dye to purple in atmospheric conditions.

PM1 Biolog plates were incubated for 24 h at 42 °C, either in the microaerophilic conditions described above or in a CampyJar with a CampyGen pack (Oxoid, Thermo Fisher Scientific, Darmstadt, Germany) at 42 °C. The atmosphere created by the CampyGen pack does not produce H${}_{2}$ and creates an atmosphere of ≈8–9% O${}_{2}$ and ≈7–8% CO${}_{2}$. Due to the availability of only one microaerophilic cabinet in the laboratory setup, a CampyGen pack was used to create a non-hydrogen condition. After incubation, both biotic and abiotic plates, in both conditions (with and without H${}_{2}$), were examined as follows:

1. Each well was examined for visual colour change due to the reduction of the Biolog Redox dye as an indication of active respiration.
2. Absorbance (OD${}_{605\mathrm{nm}}$) was measured for each well at 24 h. Absorbance at OD${}_{605\mathrm{nm}}$ captures the effect of both bacterial OD measurements and the reduced Biolog Redox dye (though the reduced form of the Biolog Redox dye absorbs maximally at 590 nm, it has an absorbance range between 400–750 nm). Therefore, the effect of substrate on bacterial cells was measured using CFU experiments, as described below.
3. A 1 μL loop was dipped into each well, and subsequently streaked onto 1/4 of an MH agar plate. These plates were incubated under standard microaerophilic conditions (as described in the Materials and Methods Section 2.1) and CFU were counted after 24 h. The maximum number of colonies counted were 200. If the streaks contained ≥200 CFU, streaks were recorded as ‘TNTC’ (Too Numerous to Count).
4. Finally, CFU were counted using serial dilutions for selected wells. These wells were selected based on a preliminary Biolog assay (examined solely for visual colour change) and GSM analysis. Substrates that indicated respiration based on colour change in our preliminary Biolog assay, and/or supported respiration in the GSM, were chosen. Substrates that didn’t support respiration, both in the preliminary Biolog assay and GSM, such as Tween 80, were also selected as a measure of negative control along with a no-carbon well. The selected wells were: no carbon source (negative control), L-aspartic acid, L-proline, L-glutamic acid, L-asparagine, L-glutamine, L-serine, succinic acid, bromosuccinic acid, L-lactic acid, formic acid, fumaric acid, D,L-malic acid, D-malic acid, L-malic acid, citric acid, acetic acid, pyruvic acid, glycolic acid, glyoxylic acid, α-hydroxy butyric acid, α-keto-glutaric acid and Tween 80. CFU were counted after 24 h.

### 2.3. Statistical Modelling

#### 2.3.1. Analysis of Absorbances

Log-transformed absorbances in the no-hydrogen condition and for H${}_{2}$ only were modelled using a separate Bayesian linear mixed model, with fixed effects of time, substrate and their interaction, and separate random effects of biological replicate at both baseline (0 h) and 24 h. Negative values were replaced with 0.01 prior to transformation. From descriptive analysis, the residual variance clearly varied with time, with much higher variation at 24 h, so the residual variance was allowed to vary with time using a distributional model. The model was estimated using brms (version 2.15.0) with rstan (version 2.26.1) using R (version 4.1.0) [36]. Four chains were estimated with 10,000 iterations per chain. A horseshoe prior with 3 degrees of freedom and 0.1 for the expected proportion of non-zero coefficients was used for the fixed effects of substrate and time; half-Student’s t-distributions with 3 degrees of freedom were used for all variance parameters. The model was validated by visual inspection of the estimated group means (substrate by time) against data points, and all diagnostic parameters, with respect to chain convergence and effective sample size, were within satisfactory ranges. Parameter estimates (the medians of posterior distributions for absorbances at both time points and the median of the absorbance ratios between baseline and 24 h) with 95% highest posterior density intervals were calculated from this model (Appendix C, Figure A2) using the emmeans package (version 1.5.5-1) [37].

#### 2.3.2. Analysis of CFU

CFU counts were analysed using a Bayesian linear mixed model, with substrate as a grouping factor. Random effects were the effect of substrate in the no-hydrogen condition (a random intercept) and the effects of H${}_{2}$ with each substrate (a random ‘slope’). An additional random effect corresponding to the individual experiment was used to account for there being up to two counts from the same biological replicate in some cases. The fixed effects were biological replicate, the average effect of H${}_{2}$, and whether the data had come from a full CFU count or the 1 μL count. Cell counts were modelled using a negative binomial distribution, with a log link, with an offset variable corresponding to the log of the volume ultimately counted (once dilutions were taken into account). Default priors were used. Data from the 1 μL experiment was treated as right-censored when values were 200 or higher (TNTC). Four chains with 5000 iterations were used for estimation. Following model estimation, the ratio of the cell concentrations between each experimental condition and the starting concentration was calculated for each iteration. Point estimates corresponding to the medians of posterior distributions of these ratios with equal-tailed 95% credible intervals were reported (Appendix C, Figure A3). Analysis was conducted using brms (version 2.15.0) with rstan (version 2.26.1) packages for R (version 4.1.0) [36].

### 2.4. Genome-Scale Metabolic Model

A GSM of *C. jejuni* M1cam developed in [8] was used in this study. The transport module of the GSM was updated to include transporters for the import of substrates present in the PM1 Biolog plate. The model includes exchange reactions for the exchange of gases present in our laboratory environment, such as O${}_{2}$, CO${}_{2}$, H${}_{2}$, inorganic substrates present in the PM1 plate, such as NH${}_{4}$, PO${}_{4}$, SO${}_{4}$ and export of by-products, such as acetate, succinate, lactate. The net stoichiometry of substrate transporters was taken from *C. jejuni* M1cam PGDB and/or MetaCyc, where possible, and belonged to different types of transport systems, such as ATP-binding cassette (ABC) transporters, the membrane electrochemical gradient energized transport and simple diffusion. For substrates for which the mechanisms of transport were not reported, the net stoichiometry was assumed to be by simple diffusion. A substrate can be transported through multiple transport mechanisms, for example, a dipeptide or oligopeptide can be transported through a proton-gradient-dependent transport system [38], or an ABC transport system [39,40]. In such cases, the import of substrates in the model was represented through multiple transport mechanisms. Note—this does not imply *C. jejuni* M1cam encodes for all these transporters, rather these transporters were included to make all substrates available to the model. Model observations were complemented with experimental results to rule out possible mis-prediction arising from the assumption.

#### 2.4.1. GSM Analysis

LP formulation, as described in Equation (1), was used to obtain an optimal flux distribution for ATP production on availability of the individual substrate.

(1) $$\begin{array}{c} \begin{array}{cc} \mathrm{minimise}: & \sum_{i=1}^{n}\left|v_{i}\cdot w_{i}\right| \end{array}\\ \mathrm{constraints}\;\;\left\{\begin{array}{c} N\cdot v=0\\ v_{ATPase}=A\\ v_{c}=0;p\leq c\leq r,c\neq q\;\left(\mathrm{see}\;\mathrm{below}\right) \end{array}\right. \end{array}$$

where v is the vector of all reaction fluxes, N is the stoichiometry matrix, and $v_{i}$ and $w_{i}$ represent the flux and weight of reaction *i* where $w_{i}=1$, unless otherwise stated. The objective function is to minimise the absolute sum of total weighted flux as a proxy for minimisation of the total enzyme investment cost [41,42,43], subject to the constraints: N×v=0 (steady-state assumption), $v_{ATPase}=A$ defines flux in the ATPase reaction (demand for ATP), and $v_{c}$ defines flux in substrate transporters with indices in range *p* to *r* which is set to zero for all nutrient transporters, except for transporters with index *q* (i.e., only one nutrient source is available, as in the experimental condition).

The LP in Equation (1) was solved for ATP production using a combination of constraints as described below:

l.1 single substrate import in the absence of H${}_{2}$: LP in Equation (1) was solved, for the production of 1 unit of ATP ($v_{ATPase}=1$), allowing import of single organic substrate (present in the PM1 plate) and constraining the import of all other substrates to 0. This analysis was performed in the absence of H${}_{2}$ (i.e., flux through H${}_{2}$ exchange reaction was set to 0), to represent the experimental condition without H${}_{2}$. A feasible solution represents GSM’s ability to produce energy on a given substrate but not necessarily biomass/growth.
1.2 with constraints on the production of biomass: LP, as described above, was solved with an additional constraint, $v_{i..j}=b_{i..j}$, to define flux through the biomass transporters, as described in [8], as a proxy for growth. A feasible solution represents GSM’s ability to produce biomass or grow on a given substrate.
1.3 in the presence of H${}_{2}$ alone: LP in Equation (1) was solved, for the production of 1 unit of ATP, allowing import of H${}_{2}$ while constraining import of all organic substrate to 0 (the experimental condition of the negative control in the H${}_{2}$ condition).

#### 2.4.2. ATP Demand Variation Analysis

ATP demand variation analysis, as described in [44], was performed to investigate the possible metabolic re-adjustment associated with changing energy demand. For this, LP in Equation (1) was solved repeatedly with an increasing demand on $v_{ATPase}$ until no feasible solution was possible (i.e., the last feasible solution represents the maximum ATP demand attainable under given constraints).

Solving Equation (1) will identify an optimal solution with the most enzyme-efficient metabolic pathways, though this could be at the expense of other resources, such as nutrients or O${}_{2}$. Given that *C. jejuni* has been isolated from nutrient-limited conditions, and that it has oxygen-labile enzymes that can be damaged by O${}_{2}$, both these factors required to be considered for analysis.

Therefore, an increased weight ($w_{O2exchange}=100$ in Equation (1)) was introduced to penalise, though not restrain, the uptake of O${}_{2}$. The substrate transporters were constrained with an upper limit ($v_{c}\leq 10$ mmoL/gDW/time) to impose a saturation on nutrient uptake. Biologically, this is a representation of saturation of transport flux which could be due to nutrient limitation and/or enzyme kinetics. Theoretically, introducing the saturation of nutrient uptake flux unravels nutrient efficient metabolic routes in an optimal LP solution, as a result, capturing the network flexibility to meet the increasing ATP demand.

#### 2.4.3. The Cost of ATP Demand

The cost of ATP demand on O${}_{2}$ utilisation, substrate utilisation and the enzyme investment cost in each of the LP solutions (in the above section) were measured as flux through the O${}_{2}$ transporter per unit of ATP produced, flux through the substrate transporter (in terms of number of carbon atoms) per unit of ATP produced, and objective value per unit of ATP produced, respectively.

All computation was achieved using the ScrumPy metabolic modelling package [45].

## 3. Results

### 3.1. PM1 Analysis

**Figure A1:**
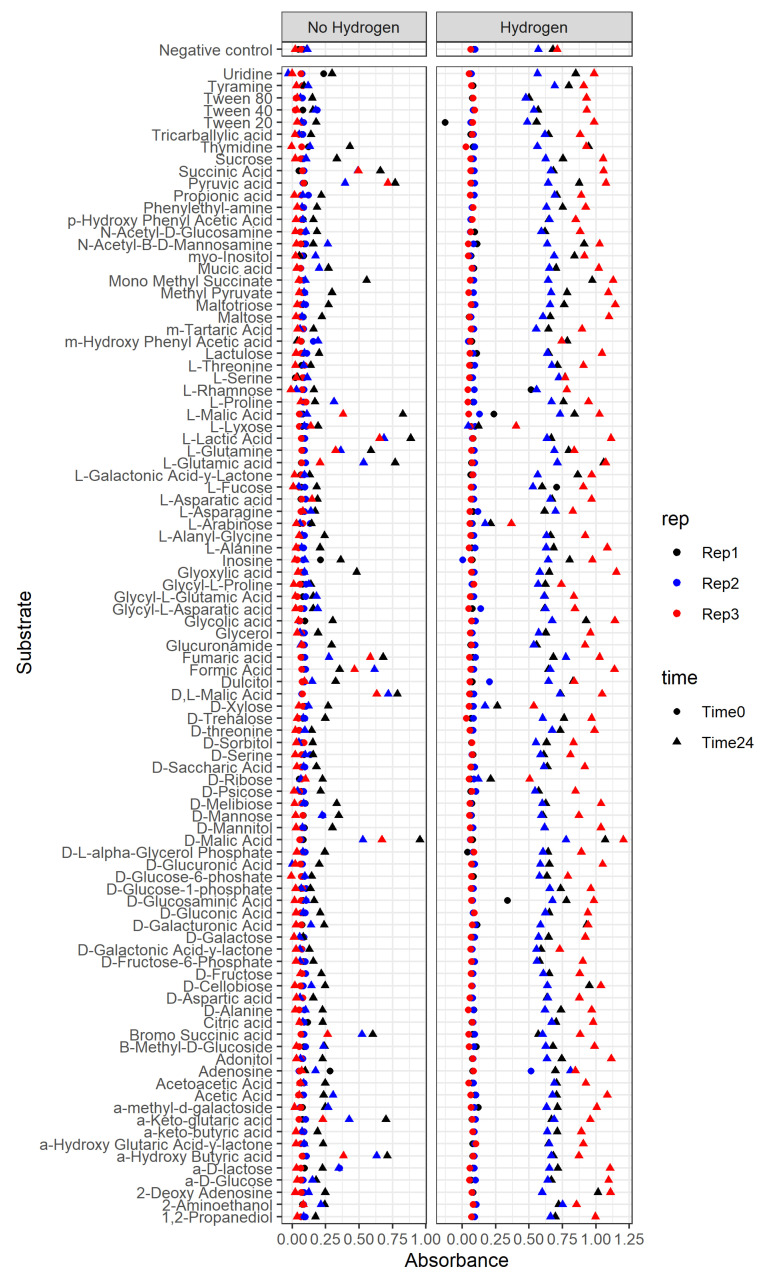
Absorbance data on individual substrate (prior to log transformation) on subtraction of background values in the abiotic plate, present in PM1 plate, with and without H${}_{2}$.

**Figure A2:**
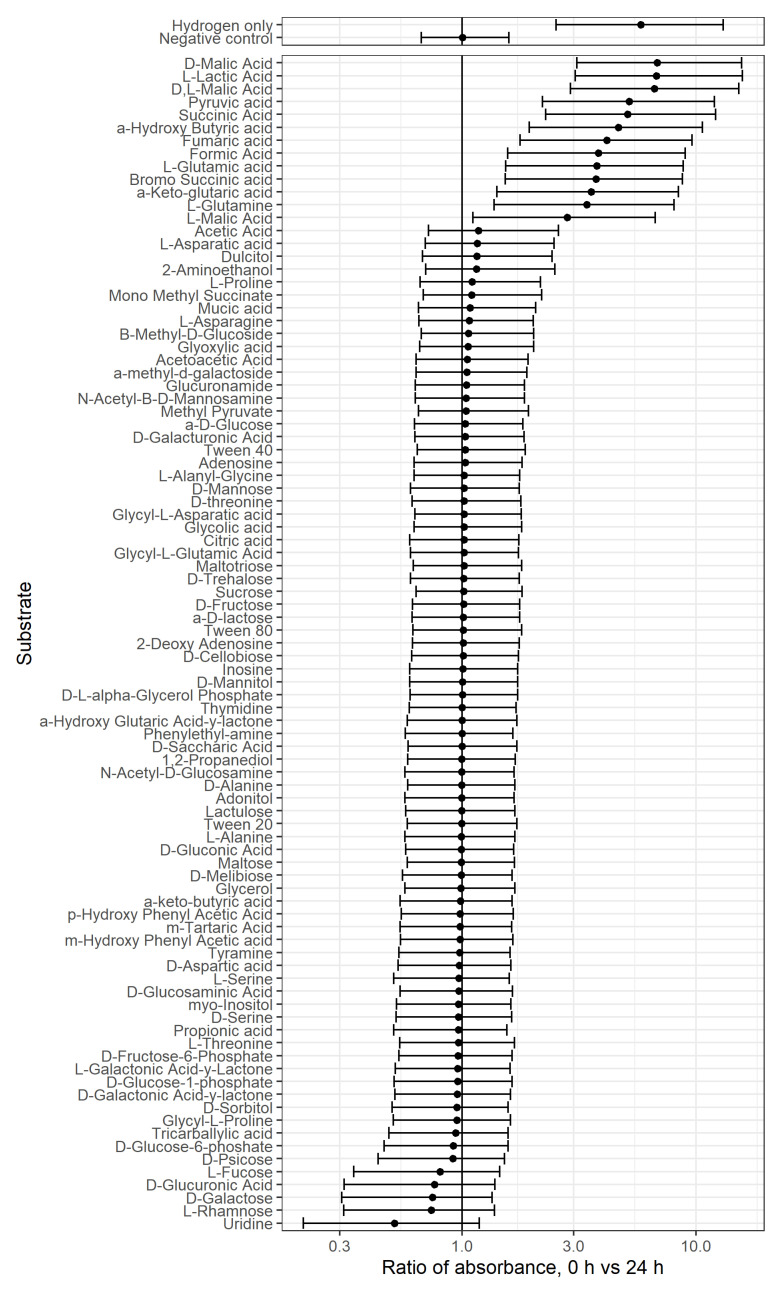
Modelled change in absorbance between 0 and 24 h incubation on each substrate under non-hydrogen conditions, compared to the negative control and H${}_{2}$ alone regarded as a single substrate. Estimates are shown with 95% high density credible intervals calculated as described in Section 2.3. Substrates are sorted on estimated absorbance change.

**Figure A3:**
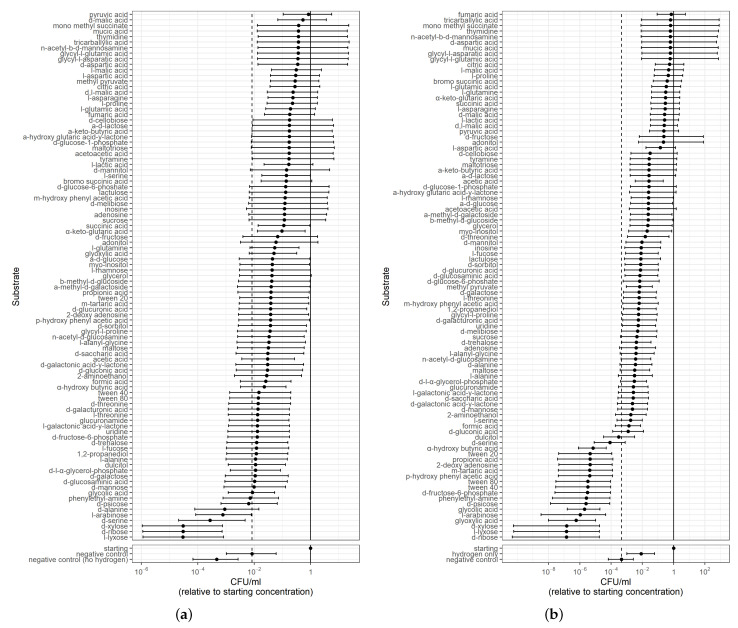
Estimated ratio of CFU/mL compared to starting concentration after 24 h incubation with each substrate (**a**) in the presence of H${}_{2}$ and (**b**) in the absence of H${}_{2}$. Dashed line in each case corresponds to the negative control (no carbon source) with/without H${}_{2}$ as appropriate. Error bars correspond to 95% equal-tailed credible intervals, as described in Section 2.3. Although, the point estimates for CFU/mL ratios are always less than 1, the possibility of slight growth due to the presence of residual auxotrophic substrates cannot be ruled out due to the upper confidence intervals. Substrates are sorted by estimated CFU/mL. CFU/mL = 1 corresponds to the starting concentration.

PM1 analysis in microaerophilic conditions, with and without H${}_{2}$, was examined for visible colour change, absorbance and CFU. Raw absorbance data on individual substrates and statistical modelling of absorbance and CFU, as described in Section 2.3, are shown in Appendix C (Figure A1, Figure A2 and Figure A3, respectively). The ratio of the CFU count at 24 h relative to the inoculum in both the conditions for all substrates was either less than or close to 1 indicating that none of the individual substrates could support cell growth. This was most likely because *C. jejuni* M1cam is auxotrophic to niacinamide, pantothenate and methionine [8], which are not available in PM1 analysis to support growth. Instead, it is likely that any colour change observed indicated respiration and represented the utilization of the substrate as a sole nutrient source for energy production, and, thus, cell survival.

#### 3.1.1. PM1 Analysis in the Presence of Hydrogen

**Figure 2:**
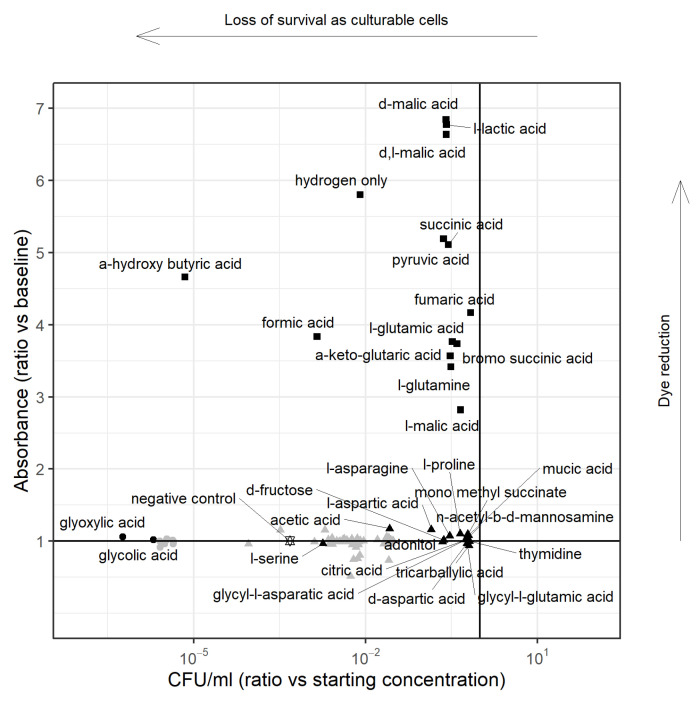
Scatterplot showing substrate utilization of *C. jejuni* M1cam. The plot shows the relationship between the modelled change in absorbance between baseline and 24 h vs. the estimated CFU/mL at 24 h relative to the starting concentration for each substrate. The shape of each marker corresponds to a clustering of the data points. Group 1 (solid square■) corresponds to substrates with visible colour change following the Biolog assay and a substantially increased absorbance during the incubation. Group 2 corresponds to substrates with no visible colour change. Using a CFU count ratio reduction of four logs split substrates into two groups. Group 2a (solid triangle ▲): supporting survival ≥ 0.0001 (▲ are the ones labelled), Group 2b (solid circle ●) not supporting survival (● are the ones labelled). All points are shown with credible intervals in the Appendix C Figure A2 and Figure A3.

PM1 analysis in microaerophilic conditions with H${}_{2}$ showed a visible colour change in all wells including the negative control (which was inoculated with bacterial cells but contained no substrate) suggesting active respiration using H${}_{2}$ alone as the substrate. The PM1 plate under the same conditions, but without bacterial inoculation (the abiotic control plate), showed no colour changes. Therefore, the possibility of H${}_{2}$ directly reducing the substrate was ruled out. Substrates under H${}_{2}$ had higher absorbance, even in the negative control, with no other substrate (Appendix C Figure A1). For the Biolog system, absorbance at OD${}_{605\mathrm{nm}}$ is a function of the colour change on reduction of the dye as well as the bacterial cell concentration, therefore cell counts were performed along with OD and colour change observations. There were some interesting discrepancies between OD and CFU for the single substrate wells (discussed below), but, in the presence of H${}_{2}$, a greater number of substrates supported relatively higher CFU (Appendix C Figure A3). However, there was no clear additive effect of H${}_{2}$ on cell survival in the presence of individual substrates when compared to the absence of H${}_{2}$. Importantly, however, the effect of H${}_{2}$ without any other nutrient source on cell survival was demonstrable; from Figure 2, it can be seen that OD${}_{605\mathrm{nm}}$ and CFU count in the presence of H${}_{2}$ alone were comparable to those in the presence of the defined substrates. Because of this, most of the wells in the H${}_{2}$ experiment were considered to contain two substrates and so were excluded from the analysis. However, H${}_{2}$ alone was regarded as a single substrate and so was compared with other substrates under non-hydrogen-containing conditions.

#### 3.1.2. PM1 Analysis for Survival on Single Substrate, including Hydrogen

As highlighted in Figure 2 as Group 1, a number of substrates with visible colour change had high absorbance ratios (absorbance at 24 h relative to the inoculum) and CFU ratios (CFU count at 24 h relative to the inoculum) around 1 showing respiration and survival as culturable cells. Three substrates stood out: for hydrogen alone, the CFU count ratio of 24 h:inoculum was 0.01, for formic acid 0.001 and for α-hydroxy butyric acid 0.00001, suggesting respiration but poor survival as culturable cells on these substrates. Using the negative control (no substrate), we defined a four-log reduction in CFU count as the cut-off for survival; α-hydroxy butyric acid was the only substrate not to support survival as culturable cells from Group 1. The substrates supporting survival were predominantly TCA cycle intermediates, amino acids, and organic acids and hydrogen.

For wells showing no colour change (Figure 2–Group 2), the CFU ratio was used to assess survival. Again, using a cut-off of a four log reduction, these substrates could be divided into two categories. Substrates that supported higher CFU counts than the negative control (Figure 2–Group 2a) included L-aspartic acid, adonitol, D-fructose, L-asparagine, L-proline, citric acid, D-aspartic acid, thymidine, tricarballylic acid, mucic acid, n-acetyl-b-d-mannosamine, mono methyl succinate, glycyl-l-glutamic acid and glycyl-l-aspartic acid, indicating cell survival, and, thus, utilisation of these substrates. On the other hand, substrates on the bottom left did not support survival (Figure 2–Group 2b), which included propionic acid, glyoxylate and glycolic acid.

### 3.2. GSM Analysis

#### 3.2.1. Substrate Utilisation

LP, as described in 1.1 of Section 2.4.1, was solved to identify substrates that could be utilised in the model for energy production.

Out of 95 substrates tested, 28 substrates (L-aspartic acid, L-serine, L-glutamine, L-proline, L-glutamic acid, L-asparagine, L-alanine, α-keto-glutaric acid, citric acid, succinic acid, fumaric acid, L-malate, formate, pyruvic acid, L-lactate, acetic acid, glycolic acid, glyoxylic acid, D-alanine, glycerol, D-fructose, D-aspartic acid, threonine, propionic acid and dipeptides (L-alanyl-glycine, glycyl-L-aspartic acid and glycyl-L-glutamic acid) were utilised in the model to support ATP synthesis. Substrates utilised in the model belonged to Group 1 and Group 2a in Figure 2, and are therefore consistent between the model and experimental observation, except for three substrates (glycolic acid, glyoxylic acid, propionic acid) that belonged to Group 2b. This discrepancy in the model was investigated, as described in Appendix B, leading to the curation of the GSM.

The same LP was solved again with additional constraints on biomass transporters, as described in 1.2 of Section 2.4.1, to test if individual substrates could support biomass production/growth in the model. No feasible solution was obtained for any of the substrate sources, suggesting that none of the individual substrates alone could support growth. Having verified the same experimentally, in Section 3.1, all further model analysis was performed focusing on energy production, without constraint on biomass production.

#### 3.2.2. Role of H${}_{2}$ on Energy Metabolism

LP, as described in 1.3 of Section 2.4.1, was solved to investigate the effect of H${}_{2}$ alone on respiration, as seen in the experimental results Section 3.1.1.

The model was able to produce ATP in the absence of any organic substrates when H${}_{2}$ was made available. Hydrogen:menaquinone oxidoreductase (hyd in Figure 1) used H${}_{2}$ to reduce the menaquinone pool that drives ETC. Complex III, Complex IV and hydrogen:menaquinone oxidoreductase contribute towards a proton gradient across the membrane which drives ATP synthesis in Complex V.

#### 3.2.3. ATP Demand Variation Analysis

ATP demand variation analysis was performed as described in Section 2.4.2.

The level of ATP demand can change the fluxes running through the interconnected metabolic network. Our analyses showed that four different mechanisms were involved in ATP production depending on substrate and demand: substrate-level phosphorylation via acetate kinase, the TCA cycle, and aerobic and anaerobic oxidative phosphorylation in the ETC. The interactions between these mechanisms are presented here using three very different substrates: glutamine (amino acid), pyruvate (a central carbon metabolite), and malate (TCA cycle intermediate). Note that there is no biomass production and, therefore, reactions involved in gluconeogenesis are inactive throughout the analysis.

**Figure 3:**
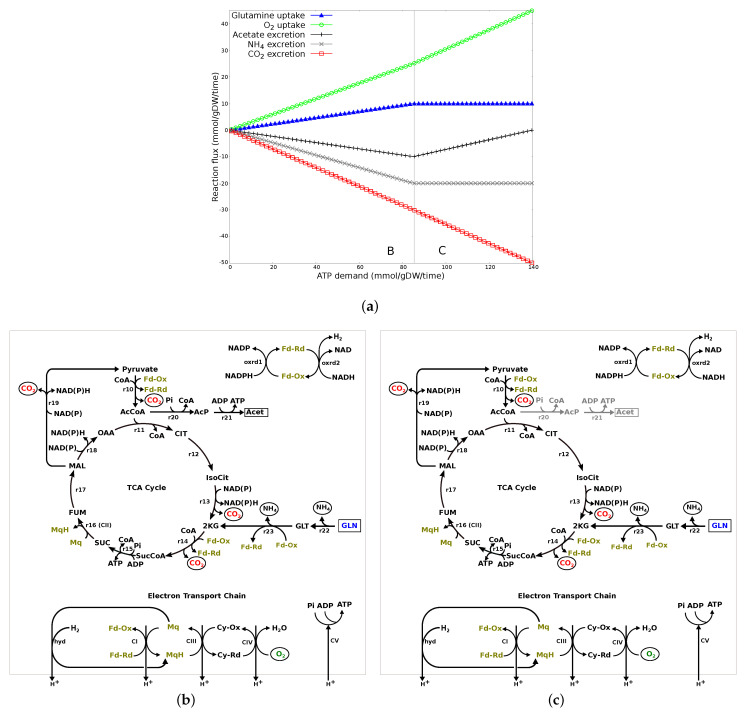
Metabolic response to changing ATP demand on glutamine. (**a**) Changes in transport fluxes with increasing ATP demand. Positive flux values represent uptake into the system while negative flux values represents excretion. The plot has been divided into two major regions labelled B and C based on the flux patterns. (**b**) Network diagram of reactions carrying flux to meet the increasing ATP demand in region B. Glutamine, on deamination (r22-r23), enters the TCA cycle through α-keto-glutaric acid. ATP demand is met through substrate-level phosphorylation through acetate kinase (r20-r21), the TCA cycle, and oxidative phosphorylation where O${}_{2}$ is used as the terminal electron acceptor. (**c**) Network diagram of reactions carrying flux to meet the increasing ATP demand in region C. Flux through acetate kinase (r20-r21) (grayed out reactions) decreases and the increasing ATP demand is met through increasing flux in the TCA cycle and aerobic oxidative phosphorylation.

Glutamine: Figure 3a shows the changes in the transport fluxes (y-axis) associated with increasing ATP demand (x-axis) when glutamine was the only nutrient source. The plot can be divided into two major flux patterns (labeled B and C) and all other reactions that varied over the range of ATP demand followed one or the other flux pattern. Figure 3b is the network diagram of all the reactions carrying flux to meet the ATP demand.

In region B, the increasing demand for ATP production is concurrent with an increase in glutamine and O${}_{2}$ uptake and excretion of acetate, CO${}_{2}$ and NH${}_{4}$. As shown in Figure 3b, glutamine, on deamination (r22, r23), enters the TCA cycle through α-keto-glutaric acid and drives the complete TCA cycle. CO${}_{2}$ and NH${}_{4}$ produced during this process are excreted out of the system, while reductants drive the oxidative phosphorylation in ETC, where O${}_{2}$ is used as a/the terminal electron acceptor. Demand for ATP is also supported through substrate-level phosphorylation, i.e., flux through phosphate acetyltransferase (r20) and acetate kinase (r21), leading to excretion of acetate.

In region C, glutamine uptake flux is at a maximum (an upper limit on nutrient uptake). Therefore, to meet the increasing ATP demand, metabolic flux is readjusted to decrease flux through acetate kinase (r20-r21 in Figure 3c) and, therefore, decrease carbon loss through acetate excretion and increase flux in the TCA cycle, O${}_{2}$ uptake and the oxidative phosphorylation in ETC.

**Figure 4:**
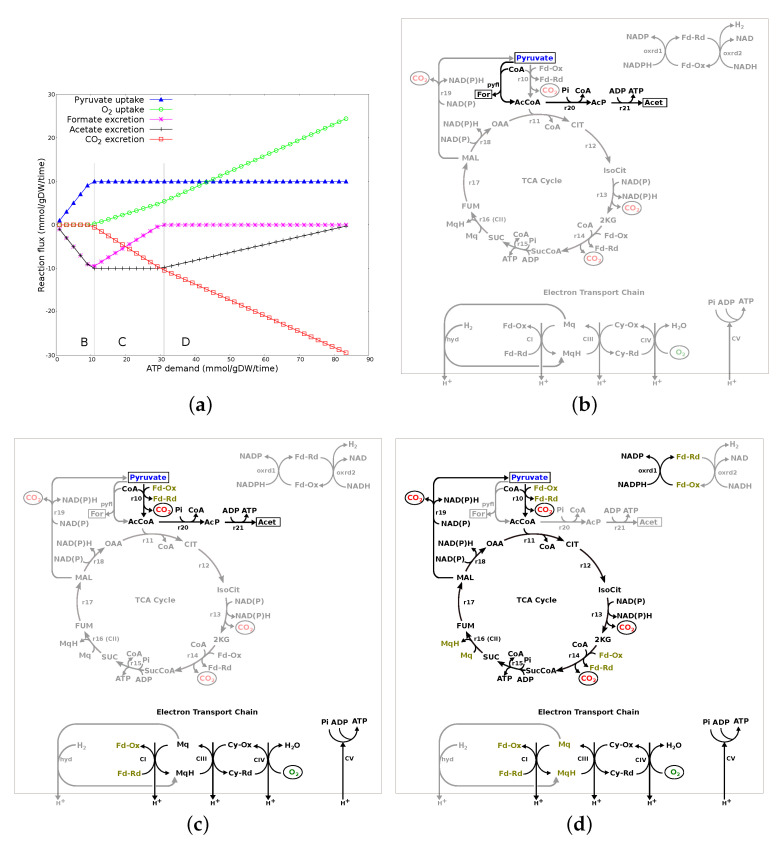
Metabolic response to changing ATP demand on pyruvate. (**a**) Changes in transport fluxes with increasing ATP demand. Positive flux values represent uptake into the system, while negative flux values represent excretion. The plot has been divided into three major regions, labelled B, C and D, based on the flux patterns. (**b**) Network diagram of reactions carrying flux to meet the increasing ATP demand in region B. ATP demand is met completely through substrate-level phosphorylation via acetate kinase (r20-r21). (**c**) Network diagram of reactions carrying flux to meet the increasing ATP demand in region C. Flux through pyruvate formate-lyase (pfl) decreases, while increasing flux in pyruvate:ferredoxin oxidoreductase (r10). ATP demand is met through substrate-level phosphorylation via acetate kinase and aerobic oxidative phosphorylation. (**d**) Network diagram of reactions carrying flux to meet the increasing ATP demand in region D. Flux through acetate kinase (r20-r21) decreases and the ATP demand is met through increasing flux in the TCA cycle and aerobic oxidative phosphorylation. Reactions that are grayed out carry zero flux (or decreasing flux where mentioned).

Pyruvate: Figure 4a shows the changes in the transport fluxes associated with increasing ATP demand when pyruvate is the only nutrient source. The plot can be divided into three major flux patterns (labeled B, C and D). Figure 4b–d represent the network diagram for each of these flux patterns.

In region B of Figure 4a, increasing ATP demand is met through increased uptake of pyruvate which is concomitant with excretion of formate and acetate. As shown in Figure 4b, pyruvate is converted to acetyl-CoA and formate by pyruvate formate-lyase (pyfl). Formate is excreted out of the system, while acetyl-CoA drives flux through acetate kinase (r20-r21) to meet the ATP demand. Note that there is no oxygen uptake in this region. The TCA cycle and ETC are inactive. ATP demand, in this region, is solely met through substrate-level phosphorylation via acetate kinase.

In region C of Figure 4a, pyruvate uptake flux has reached a maximum. The increasing ATP demand results in an increase in O${}_{2}$ uptake and CO${}_{2}$ excretion, but with a concurrent decrease in formate excretion while maintaining acetate excretion. This is explained in Figure 4c as flux, though acetate kinase is still active, thus, maintaining acetate excretion. However, precursor acetyl-CoA is generated through pyruvate:ferredoxin oxidoreductase (r10) while decreasing flux through pyruvate formate-lyase (pfl), therefore, leading to decreasing formate excretion. CO${}_{2}$ produced through pyruvate:ferredoxin oxidoreductase (r10) is excreted out of the system, while reductants (reduced ferredoxin/flavodoxin) are utilised to drive the oxidative phosphorylation in ETC, with O${}_{2}$ used as the terminal electron acceptor. ATP demand, in this region, is met through substrate-level phosphorylation via acetate kinase (r20-21) and aerobic oxidative phosphorylation without an active TCA cycle.

In region D of Figure 4a, the increasing ATP demand results in further increase in O${}_{2}$ uptake and CO${}_{2}$ excretion, while decreasing acetate excretion. As shown in Figure 4d, pyruvate, on conversion to acetyl-CoA (r10), enters the TCA cycle for complete oxidation, while flux through acetate kinase (r20-r21) decreases, leading to decrease in acetate excretion. CO${}_{2}$ produced during the process is excreted out of the system, while reductants are utilised to drive the oxidative phosphorylation in ETC, with O${}_{2}$ used as a terminal electron acceptor. ATP demand, in this region, is met through the increasing flux in the TCA cycle and aerobic oxidative phosphorylation.

**Figure 5:**
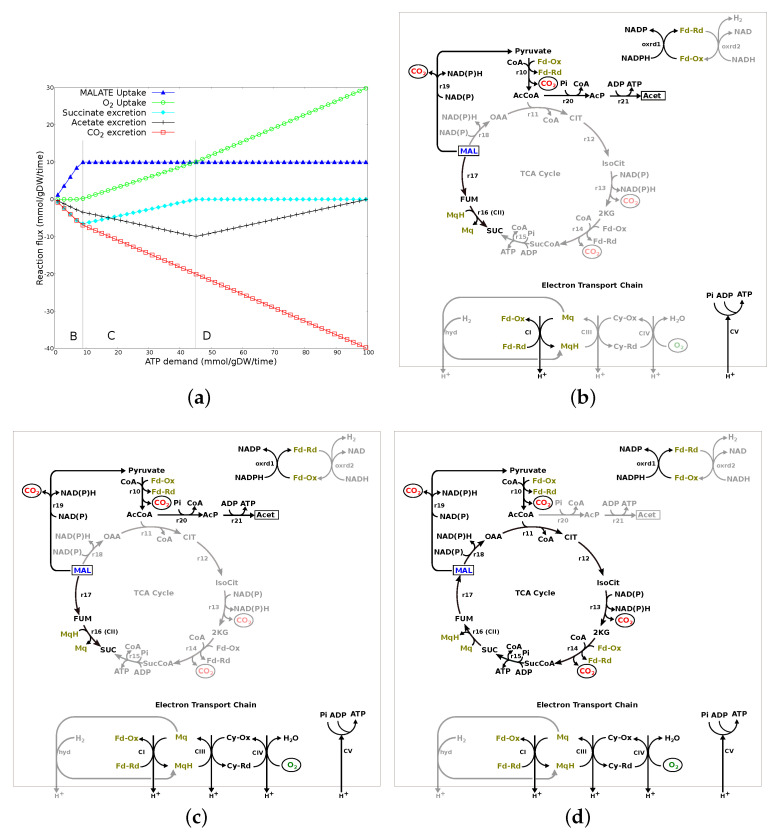
Metabolic response to changing ATP demand on malate. (**a**) Change in transport fluxes with increasing ATP demand. Positive flux values represent uptake into the system while negative flux values represent excretion. The plot has been divided into three major regions, labelled B, C and D, based on the flux patterns. (**b**) Network diagram of reactions carrying flux to meet the increasing ATP demand in region B. ATP demand is met through substrate-level phosphorylation via acetate kinase and anaerobic oxidative phosphorylation where fumarate is used as the terminal electron acceptor. (**c**) Network diagram of reactions carrying flux to meet the increasing ATP demand in region C. Flux through fumarate reductase (r16/CII) decreases, decreasing anaerobic oxidative phosphorylation, while increasing flux through acetate kinase and aerobic oxidative phosphorylation, where O${}_{2}$ is used as the terminal electron acceptor, to meet the ATP demand. (**d**) Network diagram of reactions carrying flux to meet the increasing ATP demand in region D. Flux through acetate kinase (r20-r21) decreases and ATP demand is met through the increasing flux in the TCA cycle and aerobic oxidative phosphorylation. Reactions that are grayed out carry zero flux (or decreasing flux where mentioned).

Malate: Figure 5a shows the changes in the transport fluxes associated with increasing ATP demand when malate is the only nutrient source. The plot can be divided into three major flux patterns (labeled B, C and D). Figure 5b–d represent the network diagram for each of these flux patterns.

As shown in region B of Figure 5a, increasing ATP demand is met through increased uptake of malate and concomitant release of CO${}_{2}$, acetate and succinate. Note that O${}_{2}$ is not consumed in this region. As shown in Figure 5b, ATP demand is met through substrate-level phosphorylation via acetate kinase and anaerobic oxidative phosphorylation, where fumarate is used as the terminal electron acceptor by fumarate reductase (r16/CII). CO${}_{2}$, acetate and succinate produced during the process are excreted out of the system.

In region C of Figure 5a, malate uptake flux is at a maximum (an upper limit on nutrient uptake). The increasing ATP demand is concurrent with an increase in O${}_{2}$ uptake and CO${}_{2}$ and acetate excretion while there is a decrease in succinate excretion. As shown in Figure 5c, ATP demand is met through substrate-level phosphorylation via acetate kinase (r20-r21) and anaerobic and aerobic oxidative phosphorylation. Flux through malate to succinate conversion (r16/CII and r17) decreases, decreasing succinate excretion. Flux through acetate kinase (r20-r21) increases, therefore, increasing acetate and CO${}_{2}$ excretion. Reductants produced during the process are utilised to drive the oxidative phosphorylation in ETC with both O${}_{2}$ and fumarate used as terminal electron acceptors.

In region D of Figure 5a, the increasing ATP demand is concurrent with an increase in O${}_{2}$ uptake and CO${}_{2}$ excretion while there is a decrease in acetate excretion. This is explained in Figure 5d as malate, on conversion to acetyl-CoA (r19 and r10), enters the TCA cycle for complete oxidation, while decreasing flux through acetate kinase (r20-r21), therefore, decreasing acetate excretion. Note that fumarate reductase (r16/CII) is active but in the direction of fumarate production, driving the complete TCA cycle. CO${}_{2}$ produced during the process is excreted out of the system while reductants are utilised to drive the oxidative phosphorylation in ETC, with O${}_{2}$ used as a terminal electron acceptor.

#### 3.2.4. Summary of Results

**Figure 6:**
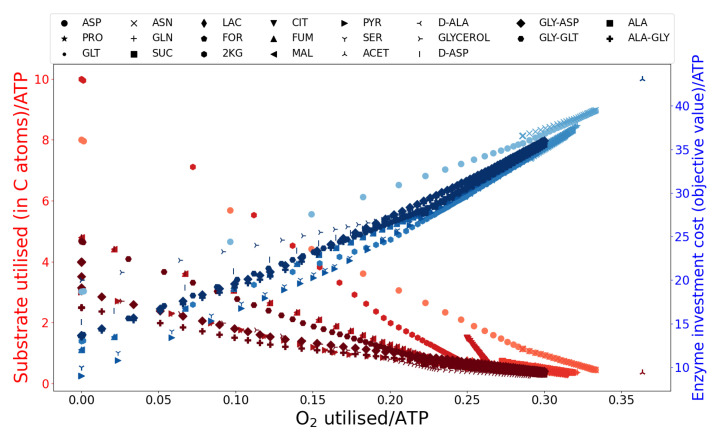
The relationship between nutrient utilisation (left y-axis, values in red), O${}_{2}$ utilisation (x-axis) and enzyme investment cost (right y-axis, values in blue) per unit ATP demand for each of the substrates utilised in the GSM.

When generating energy for survival, but not growth, *C. jejuni* M1cam can use a variety of substrates and a variety of metabolic routes to generate ATP. As shown in Table 1, the benefit of ATP production through each of the mechanisms or interactions between these mechanisms, substrate-level phosphorylation, the TCA cycle, aerobic and anaerobic respiration, brings the cost-benefit associated with nutrient availability/utilisation, O${}_{2}$ availability/utilisation and enzyme investment. This relationship is evident in Figure 6, where O${}_{2}$-independent energy production, through substrate-level phosphorylation and anaerobic respiration, though having lower enzyme investment cost, comes at the expense of a high nutrient requirement. However, O${}_{2}$-dependent energy production through aerobic respiration, though having a higher enzyme investment cost, brings the benefit of higher energy yield (energy produced per substrate) making the mechanism favourable in nutrient-limited conditions.

## 4. Discussion

*C. jejuni* M1cam can use organic acids, TCA cycle intermediates, amino acids and dipeptides, as single substrates for energy production, but not biomass production, via multiple metabolic routes. This is of practical importance because organic acids, such as malic acids, lactic acids and acetic acids, are commonly used as acidulants and marination ingredients for meat and are therefore permitted food additives that can also be used as food preservatives. They have bactericidal effects on *C. jejuni* [46,47,48,49] and some are being investigated as disinfectants in poultry slaughterhouses [50]. However, the bactericidal effect of these substrates is concentration-, pH-, and temperature-dependent. In this study, we show that, instead of having a bactericidal effect, these substrates could be providing a nutrient source and, hence, be promoting *Campylobacter* survival, at least under neutral pH conditions at temperature 42 °C. The temperature for survival in the environment will be lower but variable, so, although 42 °C is not directly representative, it was used in our experimental setup to allow comparison in conditions for which we are confident of reproducible results. We have performed experimental and computational investigations to expand the known flexibility of *Campylobacter* metabolism from biomass production to survival without growth.

All the substrates from Group 1 in Figure 2 showed a visible colour change and had high absorbance ratios. Since there was no growth, the increased absorbance on these substrates was the result of reduced tetrozolium dye rather than increase in bacterial OD (Section 2.2). Tetrazolium dye is primarily reduced by NADH and is affected by intracellular NADH concentration [51]. Therefore, it is likely that differences in absorbance within this group of substrates was due to differences in the intracellular NADH concentration and/or difference in the type of reductants involved in metabolism of these substrates and their ability to reduce the dye, as *Campylobacter* is known to utilise alternate reductants, such as flavodoxin/ferredoxin [21,22,24]. There were a number of substrates (Group 2a in Figure 2), including central metabolites and amino acids, such as L-aspartic acid, L-asparagine, L-proline, citric acid, that could support bacterial survival but were not able to reduce the tetrazolium dye. We speculate that this could be the effect of an insufficient amount of intracellular reductant, in particular NADH, and/or the inability of reductants involved in metabolism of these substrates to reduce the tetrazolium dye. The GSM was analysed for its network structural properties without taking into consideration the enzyme kinetics. Therefore, we were not able to investigate the intracellular concentration of reductants from the model. However, the network structural properties (Figure 3, Figure 4 and Figure 5) show that there can be multiple metabolic routes for utilisation of substrates with varied involvement of reductant types.

From the GSM analysis, we observed that *C. jejuni* M1cam had the metabolic flexibility to generate ATP through substrate-level phosphorylation via acetate kinase, the TCA cycle, and aerobic and anaerobic oxidative phosphorylation in the ETC. Molecular oxygen is a preferred, and also the most efficient, terminal electron acceptor for oxidative phosphorylation in *C. jejuni* [23,32], but it is not the only one. Our analyses reached the same conclusion based on a mathematical approach. O${}_{2}$ is not essential for energy metabolism, though it is essential for growth (biomass synthesis) [8,23]. *C. jejuni* is sensitive to high oxygen tension; it explicitly produces reactive oxygen species that can damage DNA, proteins, etc. Additionally, the genome encodes for oxygen-labile iron-sulphur-cluster-containing enzymes, such as PFOR and KGOR. *C. jejuni* M1cam has the flexibility to generate ATP, independent of O${}_{2}$, through substrate-level phosphorylation and anaerobic respiration, bringing an advantage in O${}_{2}$-limited, but nutrient-rich, environments, such as food storage conditions or the gut lumen.

Both, our in silico and in vitro results showed that *C. jejuni* M1cam has the ability to utilise H${}_{2}$ as an energy source. The genome encodes several hydrogenase enzymes, both soluble and membrane bound, and the ability to utilise H${}_{2}$ might bring a competitive advantage to its survival or niche adaptation in the gut environment which is known to contain H${}_{2}$-producing bacteria [52,53].

Previous studies have suggested all energy demands in *Campylobacter* can be met through oxidative phosphorylation in the ETC [24,54]. A later study confirmed that *Campylobacter* can generate ATP through substrate-level phosphorylation via acetate kinase [32]. Here, we reach the same conclusion based on our model analysis—that substrate-level phosphorylation via acetate kinase can be active alongside oxidative phosphorylation to support ATP demand.

*C. jejuni* can utilise substrates such as fumarate and nitrate as alternative terminal electron acceptors [32]. In this study, the only terminal electron acceptor provided externally was O${}_{2}$ and yet, despite O${}_{2}$ availability, our analysis shows active anaerobic respiration with fumarate was generated internally and utilised as the terminal electron acceptor. Fumarate reductase can play a key role both in aerobic and anaerobic respiration. In aerobic respiration, fumarate reductase is active in the forward direction (oxidation of succinate to fumarate) to drive the TCA cycle. However, during anaerobic respiration the activity of this enzyme is in the reverse direction utilising fumarate as an alternative electron acceptor to O${}_{2}$. Experimentally, fumarate reductase has been shown to be essential for full host colonisation [27], possibly because of its role in energy metabolism.

The same mechanisms for energy production, substrate-level phosphorylation through acetate kinase and anaerobic respiration through fumarate reductase under O${}_{2}$ limited conditions and aerobic respiration under O${}_{2}$ adequate conditions, have been suggested through transcriptomic and proteomic studies [55]. The work presented here confirms these findings through a completely independent mathematical approach.

## 5. Conclusions

*C. jejuni* is metabolically flexible and the flexibility extends to energy production for survival. The maintenance of such a variety of substrate utilisation and ATP production mechanisms suggests a selective advantage in the ability to colonise and/or survive in a wide range of habitats.

## Figures and Tables

**Table 1:** Flexibility in substrate utilisation and ATP production mechanisms in *C.jejuni* M1cam GSM, along with the range (minimum and maximum values) for substrate uptake (in terms of C atoms) per unit ATP demand, and O${}_{2}$ uptake per unit ATP demand and enzymatic cost (objective value) per unit ATP demand on individual substrate.

Substrates Utilised	Substrate-Level Phosphorylation via Acetate Kinase	Anaerobic Respiration via Fumarate Reductase	Aerobic Respiration	TCA Cycle	Nutrient Cost (Substrate Uptake/ATP)	O_2_ Uptake/ATP	Enzymatic Cost (Objective Value/ATP)
Hydrogen	×	×	✓	×	NA	0.33	38.33
Formate	×	×	✓	×	0.5	0.25	30.75
Acetate	×	×	✓	✓	0.36	0.36	43.09
Succinate	✓	×	✓	✓	0.37–0.73	0.27–0.32	32.45–37.21
Fumarate	✓	✓	✓	✓	0.4–4.8	0.0–0.3	12.0–35.57
Malate	✓	✓	✓	✓	0.4–4.8	0.0–0.3	12.0–35.57
Lactate	✓	×	✓	✓	0.32–0.75	0.25–0.32	29.75–36.76
α-keto-glutarate	✓	×	✓	✓	0.37–2.0	0.2–0.3	24.6–34.67
Citrate	✓	×	✓	✓	0.4–1.5	0.25–0.3	31.25–35.49
Pyruvate	✓	×	✓	✓	0.36–3.0	0.0–0.29	9.0–34.63
Glycerol	✓	✓	✓	✓	0.25–3.0	0.0–0.29	20.0–34.6
Proline	✓	×	✓	✓	0.29–0.42	0.29–0.31	33.96–36.45
Glutamate	✓	×	✓	✓	0.36–0.59	0.29–0.32	34.88–37.67
Glutamine	✓	×	✓	✓	0.36–0.59	0.29–0.32	35.0–37.74
L-aspartate	✓	✓	✓	✓	0.45–8.0	0.0–0.33	18.67–39.43
D-aspartate	✓	✓	✓	✓	0.4–4.8	0.0–0.3	15.2–35.67
Asparagine	✓	×	✓	✓	0.45–1.14	0.29–0.33	36.57–39.54
Serine	✓	×	✓	✓	0.36–3.0	0.0–0.29	10.0–34.75
D-Alanine	✓	×	✓	✓	0.3–1.2	0.2–0.3	27.4–35.47
L-Alanine	✓	×	✓	✓	0.3–0.67	0.22–0.3	27.78–35.57
ALA-GLY	✓	×	✓	✓	0.33–2.5	0.0–0.3	13.5–35.96
GLY-ASP	✓	✓	✓	✓	0.4–3.0	0.0–0.3	13.67–36.02
GLY-GLT	✓	×	✓	✓	0.35–7.0	0.0–0.3	20.0–35.65

where objective value is the absolute sum of total flux in Equation (1) (as described in Section 2.4.1). The values in the columns represent the ratio of flux (in substrate uptake flux, O_2_ uptake flux and total flux, respectively) to flux in total ATP production. Range of values denotes the metabolic flexibility (more than one metabolic mode) associated with individual substrate utilisation for ATP production while a number (instead of range as in case of H_2_, formate and acetate) indicates that the substrate can be utilised through a metabolic route under given conditions. Variation in O_2_ uptake/ATP, on individual substrates, are an indicator of O_2_-(in)dependent substrate utilisation and ATP production feasible at steady-state, i.e., substrates with range starting at 0 denote that the organism has themetabolic ability to utilise the individual substrate for energy production under anaerobic conditions. ✓denotes that themechanismis active while × denotes that themechanismis inactive.

## Data Availability

The data that support the findings of this study are available, on request, from https://gitlab.com/singhdi/campy-biolog (accessed on 29 June 2022). The updated GSM has also been deposited to the BioModels repository https://www.ebi.ac.uk/biomodels/MODEL2207010001 (accessed on 29 June 2022).

## Appendix A. Genome-Scale Metabolic Models Overview

A GSM describes the networks of reactions annotated in the genome of a given organism from an annotated genome. GSMs allow the investigation of the whole metabolic system, rather than individual reactions or pathways, identifying and establishing mechanistic links between genotype and phenotype. They have proved to be powerful, cost and time-effective tools for systems-level/cellular studies and have been widely applied to investigate metabolic properties, including substrate utilisation and auxotrophies, gene essentialities, and to derive hypotheses and define strategies for experimental design, and interpretation of experimental observations [56,57,58,59]. A recently published GSM for *C. jejuni* M1cam has also been successfully used to define minimal growth requirements and design minimal media [8].

Most GSMs are analysed for their network structural properties without taking into consideration the enzyme kinetics. The linear programming (LP)-based approach, a popular structural method for GSM analysis, allows the obtaining of an optimal flux distribution in the network for an objective function with given constraints. A variation of this method, introduced by [44,60], involves solving the LP problem repeatedly with incremental changes to one or more constraints. This enables relating changes in metabolic responses to changing constraints. In this study, we have used this approach to relate the change in metabolic responses to the changing ATP demand on individual substrates in *C. jejuni* M1cam.

## Appendix B. GSM Curation and Refinement from Experimental Observation

There were three substrates (glycolic acid, glyoxylic acid, propionic acid) that had a negative effect on the survival of *C. jejuni* M1cam in our experimental results (Figure 2–Group 2b), while GSM analysis suggested that these substrates could be utilised for energy metabolism. In order to identify fallacious reactions in the GSM responsible for this heterotrophy, solutions to Equation (1) on each of these substrates were examined for reactions with no gene-protein reaction association, i.e., a gene encoding the enzymes for catalysing the reaction was absent. Rather, these reactions are included based on pathway prediction algorithms and bioinformatics tools, as described in [8].

LP solutions for glycolate and glyoxylate utilisation included reactions involving isocitrate lyase and malate synthase, while, for propionic acid utilisation, the solution included methyl-isocitrate synthase. These reactions have no associated genes. Further, BlastP analysis showed that these genes were not encoded by the *C. jejuni* M1cam genome, consistent with previous reports [17,61]. Removal of these over-predicted reactions, with no GPR association, brings the model behaviour into alignment with our experimentally observed behaviour.

Conversely, there were substrates, such as thymidine, tricarballylic acid, mucic acid, and n-acetyl-b-d-mannosamine, that supported higher CFU counts in our experimental data (Figure 2–Group 2a). However, utilisation of these substrates was unpredictable from the GSM due to missing functional annotation, and thus missing metabolic routes—this was especially true for the catabolism of secondary metabolites. Enzyme annotations provide critical starting points for a GSM. Though the model used in this study provides a good coverage of central metabolism, it reflects the gap in metabolic annotation of secondary metabolism and the need for better quality functional annotations and experimental validation which is beyond the scope of the current contribution.
